# Ten-Year Outcomes of Patients with Rectal Cancer Remaining Lymph Node Positive After Preoperative Radiochemotherapy

**DOI:** 10.3390/cancers18111686

**Published:** 2026-05-22

**Authors:** Sigmar Stelzner, Stefan Niebisch, Erik Puffer, Joerg Zimmer, Dorothea Bleyl, Anja Willing, Thomas Kittner, Philipp Rhode, Matthias Mehdorn, Soeren Torge Mees

**Affiliations:** 1 Department of Visceral, Transplant, Thoracic, and Vascular Surgery, University Hospital of Leipzig, Liebigstr. 20, D-04103 Leipzig, Germany; 2 Department of General and Visceral Surgery, Dresden-Friedrichstadt General Hospital, Teaching Hospital of the Technische Universität Dresden, Friedrichstr. 41, D-01067 Dresden, Germany; 3 Institut of Pathology, Dresden-Friedrichstadt General Hospital, Teaching Hospital of the Technische Universität Dresden, Friedrichstr. 41, D-01067 Dresden, Germany; 4 Department of Radiation Oncology, Dresden-Friedrichstadt General Hospital, Teaching Hospital of the Technische Universität Dresden, Friedrichstr. 41, D-01067 Dresden, Germany; 5 Department of Medical Oncology, Dresden-Friedrichstadt General Hospital, Teaching Hospital of the Technische Universität Dresden, Friedrichstr. 41, D-01067 Dresden, Germany; 6 Department of Radiology, Dresden-Friedrichstadt General Hospital, Teaching Hospital of the Technische Universität Dresden, Friedrichstr. 41, D-01067 Dresden, Germany

**Keywords:** rectal cancer, neoadjuvant radiochemotherapy, non-response, lymph node positivity, long term outcome

## Abstract

**Background:** Persistent lymph node metastases after neoadjuvant radiochemotherapy (RCT) for locally advanced rectal cancer indicate poor response to treatment. This study evaluated the long-term prognosis of patients with residual nodal disease following neoadjuvant RCT and total mesorectal excision (TME) in comparison with patients who underwent upfront TME without adjuvant radiotherapy. **Methods:** Consecutive patients with rectal adenocarcinoma and histopathologically confirmed lymph node metastases after TME were identified from the prospectively maintained database of the colorectal unit at Dresden-Friedrichstadt General Hospital. Patients with distant metastases, in-hospital mortality, or postoperative radiotherapy were excluded. The two groups were comprehensively compared regarding patient-, tumor-, and treatment-related characteristics. Cumulative local recurrence, time to recurrence, cancer-specific survival, and overall survival were analyzed using the Kaplan–Meier method. **Results:** Between 1996 and 2021, 155 eligible patients were identified, including 101 patients in the RCT group and 54 in the upfront surgery group. Baseline characteristics were largely comparable, except for a higher median age (70.5 vs. 64 years, *p* < 0.001) and a higher proportion of lymphovascular invasion (36.0% vs. 15.2%, *p* = 0.004) in the upfront surgery group. Ten-year local recurrence rates were similar between groups (21.0% [95% CI: 10.4–31.6] vs. 20.8% [95% CI: 8.5–33.1], *p* = 0.609). No significant differences were observed in time to recurrence or cancer-specific survival. Overall survival was lower in the upfront surgery group, most likely reflecting the substantially higher age of these patients. **Conclusions:** Despite more intensive treatment, patients with a persistent ypN-positive category have outcomes no better than untreated patients with node-positive disease after TME, indicating a biologically high-risk subgroup. Non-response is therefore a sign of a negative selection. These patients may lose the opportunity for optimal local tumor control during prolonged neoadjuvant treatment, underscoring the urgent need for reliable predictive markers to identify non-responders and guide individualized treatment strategies.

## Simple Summary

Persistent metastatic lymph nodes following preoperative (neoadjuvant) radiochemotherapy (RCT) for rectal cancer represent an objective marker of treatment resistance. The long-term clinical course of these patients remains poorly characterized. In this study, we analyzed recurrence patterns and survival outcomes in patients with persistent nodal disease after neoadjuvant RCT and compared them with a cohort of node-positive patients who underwent upfront surgery without neoadjuvant treatment for various reasons. Although local recurrences occurred later in the RCT group, cumulative local recurrence rates were ultimately comparable between groups. Time to recurrence and cancer-specific survival appeared somewhat more favorable after RCT, whereas overall survival was higher but likely confounded by an approximately 10-year age difference between the cohorts. Our findings suggest that patients with persistent lymph node metastases after neoadjuvant RCT do not achieve better oncologic outcomes than node-positive patients treated without neoadjuvant therapy. Reliable predictive markers capable of identifying these lymph node-positive non-responders and guiding individualized treatment adaptation are urgently needed.

## 1. Introduction

Cure for patients with locally advanced rectal carcinoma (LARC) remains challenging, even in the era of total mesorectal excision (TME)-based surgery. The role of preoperative (neoadjuvant) radiochemotherapy (RCT) has been extensively investigated over recent decades. Both short-course radiotherapy and long-course RCT have been shown to reduce local recurrence rates by approximately 50%; however, their impact on long-term survival remains uncertain [1,2]. Total neoadjuvant therapy (TNT) was introduced to overcome this limitation and has demonstrated improvements of approximately 6 percentage points in overall survival [3] and 7 percentage points in disease-related treatment failure [4]. However, these benefits may come at the expense of increased local recurrence rates, as observed in the TNT arm of the RAPIDO trial [5]. More recently, intensified TNT strategies have been explored, showing feasibility and encouraging preliminary results [6].

In studies evaluating preoperative R(C)T, stratification of patients into responders and non-responders consistently demonstrated significantly better oncologic outcomes among responders. Response to neoadjuvant treatment was identified as an independent favorable prognostic factor in these studies [7,8,9,10,11]. Nevertheless, approximately 30% of patients continue to harbor metastatic lymph nodes after therapy and therefore remain in stage III disease [12,13]. The long-term clinical course of these patients has not been well characterized. The aim of the present study was to analyze the long-term outcomes of patients with persistent lymph node positivity after neoadjuvant treatment and to compare them with those of node-positive patients who underwent upfront surgery without adjuvant radiotherapy over a follow-up period of ten years.

## 2. Patients and Methods

Patient data were retrieved from a prospectively maintained database of the Department of General and Visceral Surgery at Dresden-Friedrichstadt General Hospital. Comprehensive preoperative staging and neoadjuvant radiochemotherapy were implemented on a larger scale beginning in 1996; therefore, the study period extended from 1996 through 2021. Ethical approval was obtained from the Ethics Committee of the Saxon Physician Chamber (EK-BR-107/25-2).

Eligible patients had histologically confirmed adenocarcinoma of the middle or lower rectum (≤12 cm from the anal verge) and underwent histopathologically complete (R0) radical resection. Exclusion criteria included synchronous metastatic disease (M1) detected during pretherapeutic staging, neoadjuvant treatment, or surgery; local excision; in-hospital mortality; and postoperative (adjuvant) radiotherapy.

All patients underwent standardized outpatient follow-up in our colorectal tumor clinic. Details of the follow-up protocol have been published previously [14]. Briefly, patients were evaluated every 6 months during the first 2 years, annually up to 5 years, and every 2 years thereafter. Follow-up investigations were performed in accordance with the German Guidelines Colorectal Cancer [15,16,17].

Preoperative staging of the primary tumor included pelvic MRI and endorectal ultrasonography. Tumors and lymph nodes were classified according to the Union for International Cancer Control (UICC) tumor-node-metastasis (TNM) classification valid at the respective time period. During the earlier study years, all visible lymph nodes were considered suspicious for metastatic involvement. Following the introduction of high-resolution MRI, additional morphologic criteria such as nodal irregularity and signal heterogeneity were incorporated into radiologic assessment [18].

Neoadjuvant radiochemotherapy was recommended for patients with clinically staged UICC stage II or III disease. During the earlier study period, only patients randomized to the treatment arm of the German CAO/AIO/ARO-94 and CAO/AIO/ARO-04 trials received preoperative RCT [19,20]. In later years, particularly after publication of the German S3 guidelines [15], all patients with LARC were considered eligible for neoadjuvant treatment. Patients deemed unfit for chemotherapy were offered short-course radiotherapy (SCRT). Only patients with an interval greater than 6 weeks between completion of neoadjuvant treatment and surgery were included. Patients who declined participation in the clinical trials or had comorbidities precluding neoadjuvant treatment, including SCRT, underwent upfront surgery.

Pelvic radiotherapy was delivered using a four-field technique extending from the promontory to the pelvic floor. Beginning in 2003, highly conformal intensity-modulated radiotherapy and Volumetric Arc Therapy techniques were introduced. Radiotherapy was administered 5 days per week in daily fractions of 1.8 Gy to a total dose of 50.4 Gy. Concurrent chemotherapy consisted of either fluorouracil (5-FU)-based treatment [19] or 5-FU combined with oxaliplatin [20]. Postoperative chemotherapy was scheduled for all patients enrolled in the CAO/AIO/ARO-94 trial or recommended after multidisciplinary tumor board discussion on an individual basis. Again, 5-FU alone or in combination with oxaliplatin was used.

Surgery was performed approximately 6–8 weeks after completion of neoadjuvant treatment and adhered to the principles of (TME) [21]. These principles were applied not only to anterior resection, but also to intersphincteric resection, Hartmann’s procedure, and abdominoperineal excision (APE). Beginning in 2006, APE was routinely performed as extralevator abdominoperineal excision according to the technique described by Holm et al. [22,23]. To account for temporal changes in treatment strategies and surgical techniques, the study period was divided into two intervals (1996–2005 and 2006–2021).

Histologically confirmed lymph node metastases after neoadjuvant treatment were considered an objective marker of treatment non-response. Tumor classification was performed according to the TNM edition valid at the respective time period. Patients who underwent upfront surgery without neoadjuvant treatment and were found to have lymph node metastases served as the control group.

Local recurrence was defined as either tumor recurrence at the anastomotic site detected endoscopically or the appearance of a new pelvic mass on imaging involving the TME-plane, locoregional lymph node stations, or the perineal scar after APE. Local recurrence rates were calculated from the date of surgery to the detection of pelvic recurrence. Cancer-specific survival and overall survival were defined as the interval from diagnosis to death from rectal cancer or death from any cause, respectively. Time to recurrence included both local and distant recurrence and was calculated from the date of diagnosis to the first documented recurrence event. Patients lost to follow-up were censored at the date of last contact, as were patients without an event at study closure (30 June 2025) or at the predefined 10-year follow-up threshold. Survival and recurrence rates were estimated using the Kaplan–Meier product-limit method. Baseline characteristics were compared using the median test, chi-square test, or Fisher’s exact test, as appropriate. To adjust for potential confounding, variables significantly differing between groups as well as neoadjuvant therapy were entered into multivariable Cox regression models for local recurrence and overall survival. A *p* value < 0.05 was considered statistically significant. Statistical analyses were performed using SPSS version 30.0 (IBM Corp., Armonk, NY, USA).

Artificial intelligence-assisted language editing was used to improve the readability and stylistic consistency of the manuscript and to correct grammar, spelling, punctuation, and syntax. Editorial support was provided using ChatGPT (OpenAI, GPT-5.5, San Francisco, CA, USA).

## 3. Results

**Figure 1:**
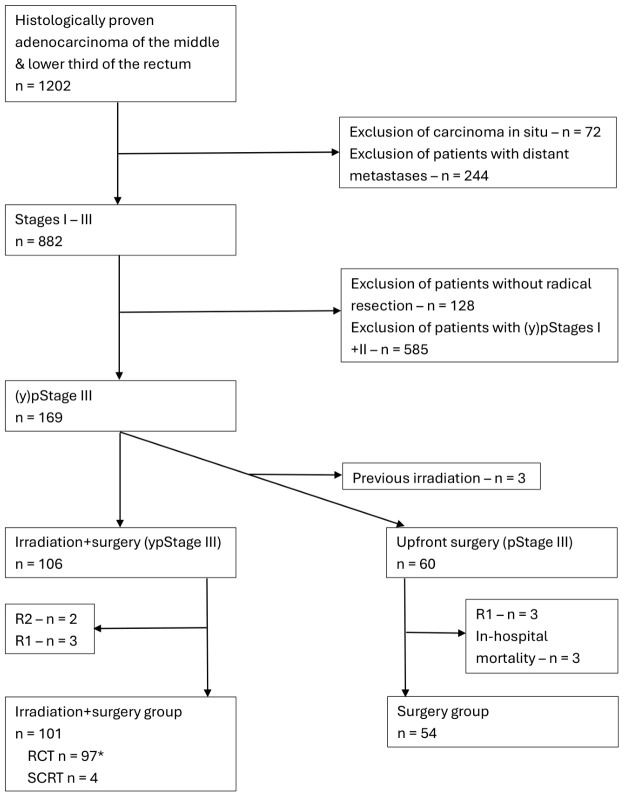
Flow chart of the study population. * one patient did not receive the full dose of radiotherapy.

Between 1996 and 2021, a consecutive series of 1199 patients with biopsy-proven rectal carcinoma was treated at Dresden-Friedrichstadt General Hospital. After application of the inclusion and exclusion criteria, 155 patients with histopathologically confirmed lymph node metastases after radical resection remained eligible for analysis, including 101 patients who had received neoadjuvant treatment and 54 who had undergone upfront surgery (Figure 1).

Persistent lymph node positivity after neoadjuvant treatment (ypTanyN1-2) was identified in 106 of 396 patients (26.8%). Five of these patients were excluded because of incomplete histopathologic resection (R1 or R2), leaving 101 patients for further analysis (neoadjuvant group). No in-hospital deaths occurred in this group. Patients with pathological stage pTanyN1-2 disease who neither received neoadjuvant treatment nor postoperative radiotherapy (*n* = 60) served as the control cohort. After exclusion of three patients with incomplete histopathologic resection (R1) and three postoperative deaths, 54 patients remained in the upfront surgery group.

Overall, the cohort comprised 99 men (63.9%) and 56 women (36.1%), with a non-significantly higher proportion of male patients in the neoadjuvant group. Patients in the surgery group were significantly older than those in the neoadjuvant group (median age 70.5 vs. 64 years, *p* < 0.001). Significantly fewer patients underwent neoadjuvant R(C)T during the earlier study period compared with the later period (16.8% vs. 83.2%, *p* < 0.001).

With respect to tumor location and clinical staging, patients in the neoadjuvant group more frequently had tumors located in the lower third of the rectum (42.6% vs. 29.6%, not significant) and presented with significantly more advanced clinical T- and N-categories. Histopathologic characteristics were generally well balanced between groups, except for lymphovascular invasion, which was significantly more common in the surgery group (36.0% vs. 15.2%, *p* = 0.004).

Notably, four patients (4.0%) in the neoadjuvant group achieved complete pathologic response of the primary tumor (ypT0), corresponding to tumor regression grade 4 according to Dworak et al. [24]. In addition, three patients in the neoadjuvant group were classified as ypN1c due to the presence of tumor deposits according to the 7th and 8th editions of the TNM classification [25,26]. Adjuvant chemotherapy was administered significantly less frequently in the surgery group than in the neoadjuvant group (18.5% vs. 64.4%, *p* < 0.001).

Median follow-up among surviving patients was 120 months and was identical in both groups. In the neoadjuvant group, six patients were lost to follow-up, although only two were lost within the first 5 years. The corresponding numbers in the surgery group were three and one, respectively. Detailed patient-, treatment-, and tumor-related characteristics are summarized in Table 1.

**Figure 2:**
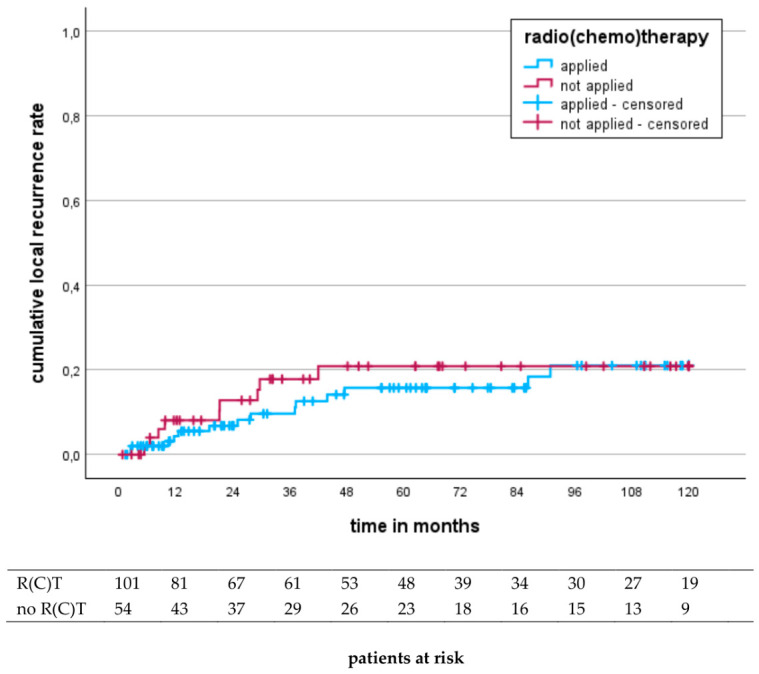
Cumulative local recurrence rates. Ten-year local recurrence rate for patients with (y)pStage III rectal cancer who received preoperative radio(chemo)therapy (R(C)T) (*n* = 101) 21.0% and for patients without R(C)T (*n* = 54) 20.8%, *p* = 0.609.

Comparison of the neoadjuvant and surgery groups revealed cumulative 10-year local recurrence rates of 21.0% (95% CI 10.4–31.6) and 20.8% (95% CI 8.5–33.1), respectively (*p* = 0.609) (Table 2, Figure 2). Median time to local recurrence was 26.4 months (range 2.4–90.8) in the neoadjuvant group and 21.1 months (range 5.3–42.0) in the surgery group (*p* = 0.400). Multivariable Cox regression analysis identified (y)pN category (N1 vs. N2; HR 2.65, 95% CI 1.08–6.50, *p* = 0.033) and lymphovascular invasion (HR 2.96, 95% CI 1.23–7.13, *p* = 0.015) as independently associated local recurrence (Table 3).

**Figure 3:**
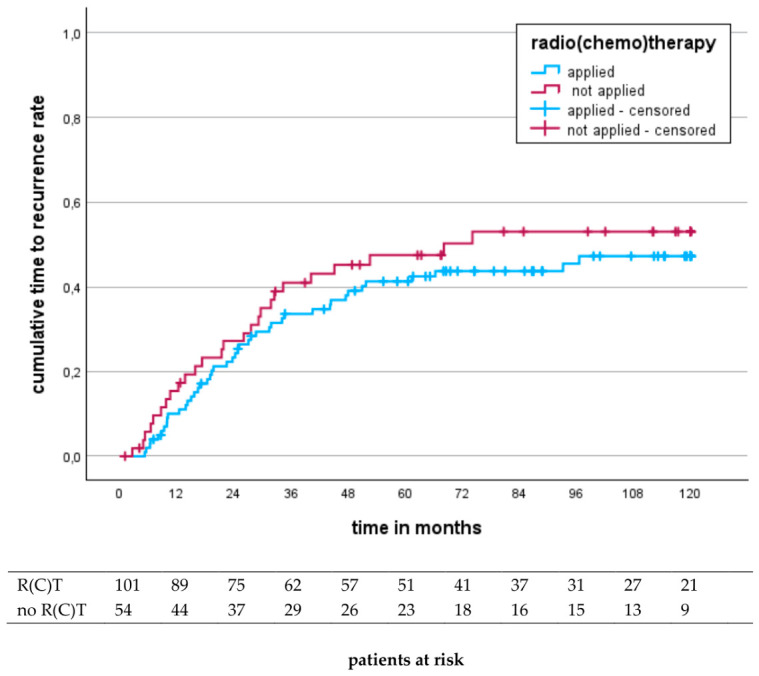
Cumulative overall recurrence rates. Ten-year time to recurrence rate for patients with (y)pStage III rectal cancer who received preoperative radio(chemo)therapy (R(C)T) (*n* = 101) 47.2% and for patients without R(C)T (*n* = 54) 53.0%, *p* = 0.419.

**Figure 4:**
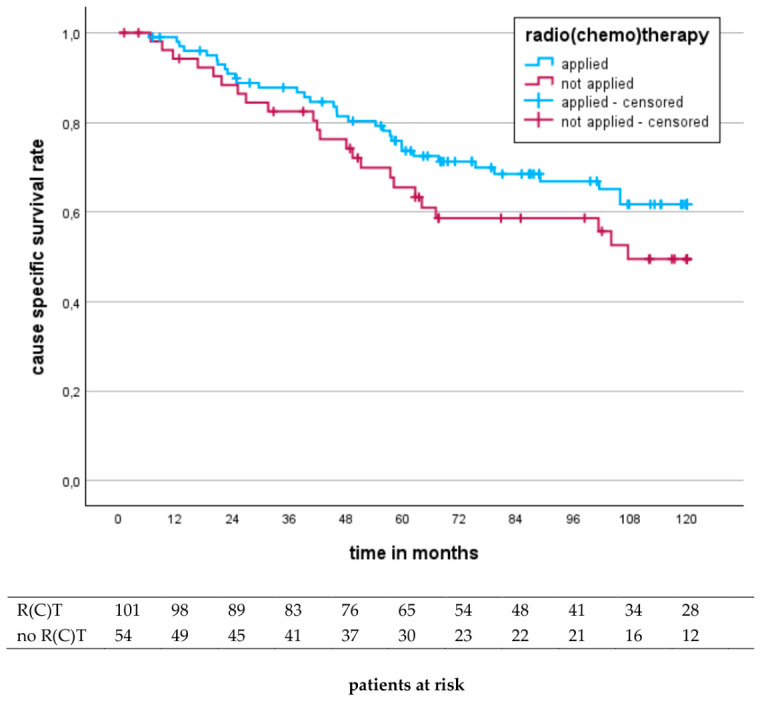
Cause-specific survival rates. Ten-year cause-specific survival rate for patients with (y)pStage III rectal cancer who received preoperative radio(chemo)therapy (R(C)T) (*n* = 101) 61.7% and for patients without R(C)T (*n* = 54) 49.5%, *p* = 0.168.

**Figure 5:**
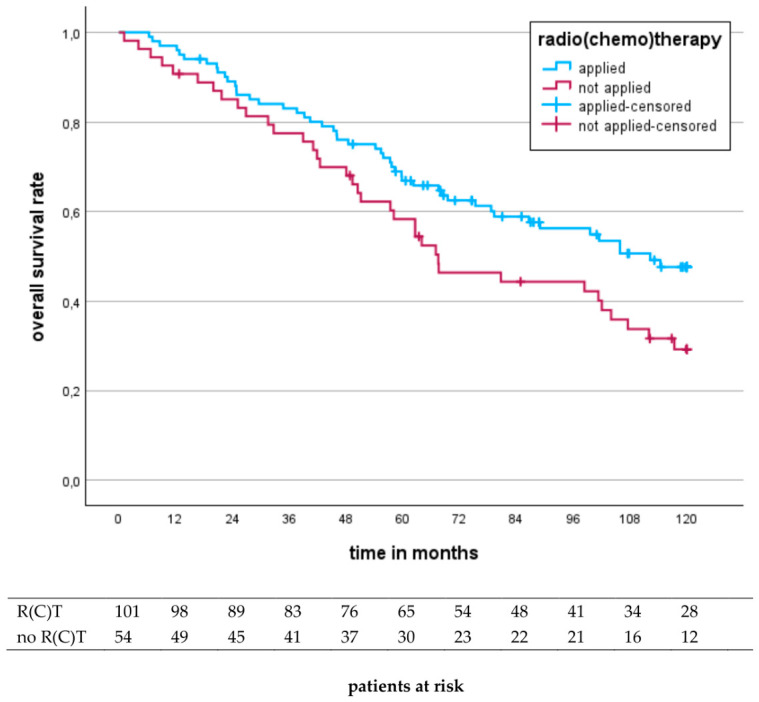
Overall survival rates. Ten-year overall survival rate for patients with (y)pStage III rectal cancer who received preoperative radio(chemo)therapy (R(C)T) (*n* = 101) 47.6% and for patients without R(C)T (*n* = 54) 29.8%, *p* = 0.037.

The cumulative 10-year time to recurrence rate (local and distant recurrence combined) was 47.2% (95% CI 36.6–57.8) in the neoadjuvant group and 53.0% (95% CI 38.7–67.3) in the surgery group (*p* = 0.419) (Figure 3). Ten-year cancer-specific survival rates were 61.7% (95% CI 50.9–72.5) and 49.5% (95% CI 34.2–64.8), respectively (*p* = 0.168) (Figure 4). As expected, overall survival at 10 years was significantly higher in the neoadjuvant group (47.6%, 95% CI 37.0–58.2) than in the surgery group (29.2%, 95% CI 16.5–41.9; *p* = 0.037) (Figure 5). Multivariable analysis confirmed age (HR 1.04 per year, 95% CI 1.01–1.06, *p* = 0.010) and (y)pN category (N1 vs. N2; HR 2.19, 95% CI 1.37–3.49, *p* = 0.001) as independently associated with overall survival (Table 4).

## 4. Discussion

Our study demonstrates that patients with persistent metastatic lymph nodes after neoadjuvant R(C)T experience local recurrence rates comparable to those of node-positive patients undergoing upfront surgery without pretreatment, thereby reflecting the natural course of node-positive rectal cancer in the TME era. Although not statistically significant, the median time to local recurrence was longer in the neoadjuvant group, with recurrences occurring beyond 5 years, a pattern not observed in the surgery group. Similarly, time to recurrence rates and cancer-specific survival did not differ significantly between the groups. The observed difference in overall survival was expected and was confirmed in multivariable analysis to be largely attributable to the substantially higher median age of patients in the surgery group.

Persistent lymph node metastases after neoadjuvant R(C)T clearly indicate absent or at least poor response to treatment and identify a subgroup of patients who may derive limited benefit from this preoperative strategy.

Residual nodal disease is observed in approximately 25% of patients after neoadjuvant radiochemotherapy compared with approximately 40% in patients treated with upfront surgery when neoadjuvant treatment is indicated on the basis of clinical stage II or III disease [19]. Comparable findings have also been reported after short-course radiotherapy followed by delayed surgery, with ypN positivity rates of 26.3% versus 34.2% after immediate surgery [27]. Results from intensified neoadjuvant approaches such as total neoadjuvant therapy (TNT), however, have been inconsistent across trials. In PRODIGE 23, intensified induction chemotherapy combined with RCT significantly reduced ypN-positive disease compared with conventional RCT (17% vs. 32%) [28]. A smaller but still significant reduction was observed in the RAPIDO trial, which compared short-course radiotherapy followed by consolidation chemotherapy with standard RCT (25% vs. 32%) [4]. In contrast, the STELLAR trial found no significant difference between short-course radiotherapy plus consolidation chemotherapy and conventional RCT (29.5% vs. 31.7%) [29]. Nevertheless, TNT remains a promising strategy for locally advanced rectal cancer because of its improved complete response rates [30].

Recently, Diefenhardt et al. analyzed pooled data from three German randomized trials to investigate the prognosis of patients with persistent nodal metastases after neoadjuvant treatment [31]. Among 1888 patients, 522 (29.2%) remained lymph node positive, including 378 patients (20.0%) with ypN1 disease and 174 patients (9.2%) with ypN2 disease. The authors demonstrated marked differences in local recurrence, distant metastasis, and survival according to nodal status. Five-year local recurrence rates were 3% for ypN0, 6% for ypN1, and 19% for ypN2 disease. Similarly, distant metastasis rates were 20%, 40%, and 72%, respectively, while overall survival rates at 5 years were 86.1%, 74.0%, and 43%. Importantly, TNT reduced the incidence of ypN2 disease (6% vs. 11.3%). The authors concluded that patients with persistent nodal disease after neoadjuvant therapy require intensified postoperative surveillance [31].

Another retrospective analysis of the German CAO/ARO/AIO-94 trial comparing preoperative versus postoperative RCT demonstrated no difference in 10-year local recurrence rates but substantially higher distant metastasis rates and worse disease-free survival among patients receiving preoperative RCT when analyzed according to identical TNM stages [32]. These findings suggest that, although preoperative RCT improves outcomes in the overall population, it may simultaneously select for a subgroup with biologically aggressive, radiotherapy-resistant tumors. Our data support the hypothesis that patients with persistent nodal positivity after neoadjuvant R(C)T have long-term outcomes comparable to node-positive patients who did not receive radiotherapy, despite undergoing more intensive multimodal treatment and receiving adjuvant chemotherapy more frequently. This suggests that persistent nodal positivity identifies a radiotherapy-resistant phenotype that may not derive the expected benefit from neoadjuvant treatment.

Particularly noteworthy is the similarity in local recurrence rates between groups, despite a delayed occurrence of recurrence after neoadjuvant treatment. This phenomenon became apparent shortly after the widespread implementation of preoperative therapy around 2005 [33]. In patients who fail to respond to radiotherapy, prolonged treatment intervals before surgery may compromise local tumor control. Consistent with this concept, patients treated in the experimental arm of the RAPIDO trial experienced significantly higher local recurrence rates than those receiving conventional RCT [5]. These findings likely reflect the unfavorable biology of non-responding tumors, which continue to progress despite intensive treatment and may ultimately lose the opportunity for complete surgical clearance because of delayed resection. Similar concerns have been raised for patients managed with watch-and-wait strategies who subsequently develop incomplete response or local regrowth [34].

Given the poor prognosis of non-responders, reliable predictive factors for persistent lymph node positivity would be of considerable clinical value. Several studies have identified age, clinical T- and N-category, residual tumor diameter, tumor grade, signet-ring or mucinous histology, perineural and lymphovascular invasion, CEA levels, and the interval between completion of RCT and surgery as factors associated with ypN-positive disease [12,31,35,36,37]. However, none of these parameters specifically predicts treatment resistance, and patient selection remains challenging. Newton et al. integrated several of these variables into a predictive nomogram; however, its accuracy reached only 70.9% [35].

The prognostic difference between patients achieving ypN0 status and those with persistent nodal involvement after neoadjuvant therapy is well established [38]. However, clinical nodal staging is limited by substantial overstaging [39]. Consequently, patients who are clinically node-positive but pathologically node-negative may artificially improve the apparent outcomes of downstaged cohorts. For this reason, several studies have evaluated nodal tumor regression as a prognostic marker. Results remain inconsistent, with some studies suggesting that nodal regression is associated with favorable prognosis in ypN0 disease [9,10,40], whereas others reported worse outcomes compared with ypN0 patients without evidence of prior nodal involvement [41,42].

The major strengths of our study include the availability of a comparison group treated with upfront surgery without postoperative radiotherapy and the exceptionally long follow-up derived from a well-maintained prospective database with predominantly direct patient contact. Nevertheless, several limitations should be acknowledged. First, the retrospective design is associated with all drawbacks inherent in this study design. Second, multiple editions of the TNM classification were in operation during the study period. Because subtle changes in the classification of small tumor nodules were not consistently documented in the database, uniform retrospective reclassification was not feasible. As this issue affected only pretreated patients, any resulting bias would likely favor the neoadjuvant group. Third, the dataset reflects routine clinical practice and lacks comprehensive molecular tumor characteristics for most patients. Despite these limitations, the present study provides valuable long-term insights into the clinical course of patients with persistent lymph node metastases after neoadjuvant RCT compared with radiotherapy-naïve node-positive patients treated with upfront surgery.

## 5. Conclusions

Persistent lymph node positivity after neoadjuvant R(C)T identifies a biologically aggressive high-risk subgroup with outcomes comparable to those of untreated node-positive patients. Reliable predictive markers are urgently needed to identify non-responders before treatment initiation and to avoid ineffective neoadjuvant therapy in these patients.

## Figures and Tables

**Table 1:** Patient, treatment, and tumor characteristics.

	Total *n* = 155	Neoadjuvant Therapy *n* = 101	Upfront Surgery *n* = 54	*p*
Age, median in years (range)	65 (29–91)	64 (29–83)	70.5 (51–91)	<0.001
Sex male female	99 (63.9) 56 (36.1)	69 (68.3) 32 (31.7)	30 (55.6) 24 (44.4)	0.115
Time period 1996–2005 2006–2021	51 (32.9) 104 (67.1)	17 (16.8) 84 (83.2)	34 (63.0) 20 (37.0)	<0.001
Distance from anal verge ≤12 cm <6 cm	96 (61.9) 58 (38.1)	58 (57.4) 43 (42.6)	38 (70.4) 16 (29.6)	0.114
Staging modalities ^a^ 1996–2005 MRI MRI + EUS EUS Others 2006–2021 MRI MRI + EUS EUS others	*n* = 51 34 (66.7) 30 (58.2) 32 (62.7) 5 (9.8) *n* = 104 97 (93.3) 31 (29.8) 37 (35.6) 1 (1.0)	*n* = 17 14 (82.4) 11 (64.7) 12 (70.6) 2 (11.8) *n* = 84 78 (92.9) 26 (31.0) 31 (36.9) 1 (1.2)	*n* = 34 20 (62.5) 19 (55.9) 30 (88.2) 3 (8.8) *n* = 20 19 (95.0) 5 (25.0) 6 (30.0) 0 -	-
Clinical T-category cT1 cT2 cT3 cT4	3 (1.9) 14 (9.0) 48 (31.0) 90 (58.1)	1 (1.0) 1 (1.0) 11 (10.9) 88 (87.1)	2 (3.7) 13 (24.1) 37 (68.5) 2 (3.7)	<0.001
Clinical N-category cN0 cN1 cN2	32 (20.6) 37 (23.9) 65 (41.9)	11 (10.9) 28 (27.7) 62 (61.4)	21 (38.9) 9 (16.7) 24 (44.4)	<0.001
Pathologic T-category ypT0 (y)pT1 (y)pT2 (y)pT3 (y)pT4	4 (2.6) 4 (2.6) 41 (26.5) 99 (63.9) 7 (4.5)	4 (4.0) 2 (2.0) 27 (26.7) 62 (61.4) 6 (5.9)	- 2 (3.7) 14 (25.9) 37 (68.5) 1 (2.9)	0.388
Pathologic N-category (y)pN1 (y)pN2	108 (69.7) 47 (30.0)	72 (71.3) 28 (28.7)	36 (66.7) 18 (33.3)	0.551
Number of retrieved lymph nodes ^b^, median (range)	13 (3–41)	13 (3–41)	14.5 (5–36)	0.365
Number of involved lymph nodes ^b^, median (range)	2 (0–19)	2 (0–11)	2 (1–19)	0.910
Lymph node ratio ^b^, median (range)	0.154 (0–1.0)	0.154 (0–0.88)	0.156 (0.03–1.0)	0.913
Grading ^b^ well/moderate poor	108 (70.6) 45 (29.4)	70 (70.7) 29 (29.3)	38 (70.4) 16 (29.6)	0.965
Pretherapeutic CEA-level ^c^ ≤5 ng/mL elevated	96 (66.2) 49 (33.8)	57 (62.0) 35 (38.0)	39 (73.6) 14 (26.4)	0.154
Lymphovascular invasion (L) ^d^ no yes	116 (77.9) 33 (22.1)	84 (84.8) 15 (15.2)	32 (64.0) 18 (36.0)	0.004
Vascular invasion (V) ^e^ no yes	125 (84.5) 23 (15.5)	82 (82.8) 17 (17.2)	43 (87.8) 6 (12.2)	0.436
Tumor regression 0 1 2 3 4	n.a.	1 (1.0) ^f^ 13 (12.9) 19 (18.8) 58 (57.4) 4 (4.0)	n.a.	-
pCRM ^g^ free involved	104 (98.1) 2 (1.9)	83 (98.8) 1 (1.2)	21 (95.5) 1 (4.5)	0.303
Intraoperative perforation no yes	141 (91,0) 14 (9.0)	94 (93.1) 7 (6.9)	47 (87.0) 7 (13.0)	0.212
Emergency procedure no stoma as emergency yes	151 (97.4) 4 (2.6) -	98 (97.0) 3 (3.0) -	53 (98.1) 1 (1.9) -	1.0
Procedure APE Anterior resection HARTMANN	35 (22.6) 111 (71.6) 9 (5.8)	26 (25.7) 72 (71.3) 3 (3.0)	9 (16.7) 39 (72.2) 6 (11.1)	0.071
Adjuvant chemotherapy no yes	80 (51.6) 75 (48.4)	36 (35.6) 65 (64.4)	44 (81.5) 10 (18.5)	<0.001
Follow-up ^h^, medina in months (range)	122.0 (12.7–174.2)	122.0 (17.1–174.2)	122.0 (12.7–158.3)	0.152

Numbers in round brackets are percentages if not otherwise specified. ^a^—in this raw, percentages are given per time period. Because of overlapping staging modalities, percentages do not sum up to 100%; ^b^—2 missing; ^c^—10 missing; ^d^—6 missing; ^e^—7 missing; ^f^—6 missing; ^g^—49 missing; ^h^—surviving patients; n.a.—not applicable.

**Table 2:** Oncological outcomes at 10 years.

	Total *n* = 155	Neoadjuvant Therapy *n* = 101	Upfront Surgery *n* = 54	*p*
Cumulative local recurrence rate events	21.1 (12.9–29.3)% 23	21.0 (10.4–31.6)% 14	20.8 (8.5–33.1)% 9	0.609
Cumulative time to recurrence rate events	49.1 (40.7–57.5)% 70	47.2 (36.6–57.8)% 44	53.0 (38.7–67.3)% 26	0.419
Cause-specific survival events	57.5 (48.7–66.3)% 56	61.7 (50.9–72.5)% 33	49.5 (34.2–64.8)% 23	0.168
Overall survival events	40.8 (32.4–49.2)% 84	47.6 (37.0–58.2)% 48	29.2 (16.5–41.9)% 36	0.037

Numbers in round brackets are 95% confidence intervals.

**Table 3:** Cox regression analysis for local recurrences.

	Hazard Ratio	95% CI	*p*
Age	1.02	0.97–1.07	0.489
Time period 1996–2005 2006–2021	Ref. 0.55	0.21–1.43	0.219
Neoadjuvant R(C)T No (upfront surgery) Yes	Ref. 0.53	0.18–1.58	0.256
Lymphovascular infiltration ^a^ No Yes	Ref. 2.96	1.23–7.13	0.015
(y)pN-Category 1 2	Ref. 2.65	1.08–6.50	0.033
Adjuvant chemotherapy No Yes	Ref. 0.58	0.22–1.57	0.287

149 patients included because of 6 missing values in (^a^).

**Table 4:** Cox regression analysis for overall survival.

	Hazard Ratio	95% CI	*p*
Age	1.04	0.01–1.06	0.010
Time period 1996–2005 2006–2021	Ref. 0.55	0.21–1.43	0.219
Neoadjuvant R(C)T No (upfront surgery) Yes	Ref. 0.81	0.45–1.44	0.474
Lymphovascular infiltration ^a^ No Yes	Ref. 1.10	0.64–1.89	0.728
(y)pN-Category 1 2	Ref. 2.19	1.37–3.49	0.001
Adjuvant chemotherapy No Yes	Ref. 0.65	0.41–1.14	0.148

149 patients included because of 6 missing values in (^a^).

## Data Availability

The raw data supporting the conclusions of this article will be made available by the authors on request.
